# Active machine learning approach to adversarial training improves trade-off between natural accuracy and adversarial robustness

**DOI:** 10.1038/s41598-026-50378-5

**Published:** 2026-04-28

**Authors:** Seyed Mohammad Hadi Mirsadeghi

**Affiliations:** 1 Department of Software Science, Tallinn University of Technology, Tallinn, 19086 Estonia; 2 Department of Mathematics, Aarhus University, Aarhus, DK-8000 Denmark

**Keywords:** Adversarial training, Active machine learning, Accuracy-robustness trade-off, Engineering, Mathematics and computing

## Abstract

Understanding our world which is open and diverse requires foundation models that generalize well while trustworthy. Adversarial training has been considered to be one of the most effective strategies to achieve robust learning systems, yet adversarial training methods have exhibited a trade-off between generalization accuracy and robustness. Motivated by the active machine learning approach to adversarial training, we introduce novel data generation technique acquires adversarial examples based on classification margin criterion, addressing key trade-off between generalization accuracy and adversarial robustness which remains a fundamental challenge in adversarial training. Here, we provide theoretical contribution that sheds light on the properties of the approach which is expected to be beneficial. Likewise, we empirically demonstrate that the proposed method achieves significant improvements in accuracy and robustness with WRN34-10 and ResNet-18 on CIFAR-10, CIFAR-100, SVHN, and TinyImagenet-200. This Article contributes to the broader scientific literature on adversarial training, generalization theory, and robust machine learning.

## Introduction

Be it owing to the fractional dimensionality of features mapped onto artificial neurons^1^ or the linearity of deep neural networks at their core^2^, adversarial examples - class of imperceptibly perturbed instances such that they result in the model outputting an incorrect answer with high confidence^2^ - unveil compelling truths about the training process and the so-called true model. This is indicated by energy-level jumps during adversarial training^1^, that is, training neural networks on regular and adversarial examples^2,3^. According to the manifold assumption, training on off-manifold examples during adversarial training leads to robust networks and training on on-manifold ones is essentially generalization^4^. Fractional dimensionality is a puzzling phenomenon where one instance may belong to more than one class at the same time. To train on such fractionally represented instances is key to achieving robust networks. To train on all such examples, however, will likely lead to a decrease in generalization accuracy.

Recent studies suggest there may be a trade-off between adversarial robustness and natural accuracy^5–8^. A recent study^8^ even suggests that robustness and generalization are conflicting goals. On the contrary, it has been argued that robust and accurate models are actually possible at the cost of computational overhead^4^. It is important to note that different models and training strategies exhibit different characteristics with respect to the relationship between robustness and generalization. A recent study^4^assumes an underlying, low-dimensional data manifold and shows that adversarial robustness and generalization are not opposing goals, i.e. both robust and accurate models are possible but require higher sample complexity^9^. [9] shows that removing some adversarial examples during adversarial training does not hurt robustness. Determining the importance of each training example can efficiently reduce the computational cost of adversarial training while preserving its robustness and improving natural accuracy.

In the passive learning framework, the learning system has access to the entire labeled dataset and uses it to approximate a predictive model. The learning process is typically divided into two steps: training and testing. The goal of passive learning is to find statistical regularities during training and approximate a function that maps inputs to desired outputs at test time. Active machine learning, however, is based on optimal experimental design^10^ which is a subfield of machine learning where the key hypothesis is that if the learning algorithm chooses the training data from which it learns, it may reach good accuracy with less data in less time^11^. There are several scenarios for active machine learning, namely membership query synthesis^12^, selective sampling^13^, and pool-based active learning^14,15^. In membership query synthesis, the learning system may query any unlabeled instance even if it is not sampled from the underlying data distribution. Selective sampling refers to the scenario where an unlabeled instance may first be sampled from the actual distribution for the learner to decide whether or not to request its label. Pool-based active learning assumes that unlabeled data may be gathered at once and queries may be selectively drawn from the pool. Active machine learning scenarios involve evaluating the informativeness of unlabeled instances. Uncertainty sampling^14^ queries the instances about which the learner is least confident as to how to label. Classification margin sampling measures the distance between the first and second most likely predictions and selects instances with lower margin values. Classification entropy sampling selects the instance with the highest entropy. The goal of active machine learning is to learn a good approximation of a concept with as few samples as possible. This goal is shared by model extraction attacks^16^ where adversary submits queries to the learner in order to understand the model. In active learning and model extraction, the acquisition function or the query interface aims to select the most informative samples in order to learn a good approximation of the underlying concept or model with as few samples as possible. Recent research suggests that some adversarial examples are less important, i.e., removing some adversarial examples from adversarial training does not negatively impact robustness^9^. Generalization and adversarial robustness are achieved by selecting a sufficient number of important data points and finding the sweet spot between under-fitting and over-fitting. Attempting to improve the generalization capability of the model with only few informative points is equivalent to asking the question: If adversary had a modest query budget to extract the model, what points would adversary query? By answering this question, one may learn the model in question with good accuracy with only few queries. Model extraction can also be viewed as a form of active machine learning where the substitute model is able to obtain labels on new points from the model under study^16^. The goal of active machine learning is to select the most informative data points for training. Acquisition function is the key factor in the success of active machine learning^17^.

Adversarial training can be viewed analogous to the game of billiards. Our goal is to make the balls in an accurate way and refrain from a scratch. The proficient player will incorporate active calibration of shots. We propose the same during adversarial training, that is, to be actively selective with the subset of adversarial examples to train on at each batch. To square the circle and train on a good subset of adversarial instances may make all the difference in addressing key trade-off between generalization accuracy and adversarial robustness. More to the point^8^, argued that robustness may be at odds with natural accuracy. We concur that this is typically the case when following common practice^3^ and training on 50% randomly selected adversarial examples during batch training. We suggest an alternative approach to training robust models. We suggest acquiring adversarial examples based on an informativeness criterion during batch training. Our main hypothesis is that it may be possible to improve both natural accuracy and robustness provided that selected adversarial examples add significant information to the model’s concept during training. Instead of learning from all adversarial examples, or a randomly selected subset of all adversarial examples, we suggest treating adversarial training as a case of active machine learning. The proposed method selects adversarial and natural examples based on classification margin. The integration of margin-based sample selection into existing adversarial training paradigms is promising and practically appealing. It has been shown^18^ that infinite data could eliminate the accuracy-robustness trade-off. Given that we can launch PGD attack^5^ with different steps and initialization points, it is possible to think of the space of all adversarial examples during batch training as an infinite set. This implies that adversarial training must learn from a subset of all adversarial examples. We take the active machine learning approach by treating adversarial training as a case of active learning. During the training process, our goal is to learn from examples with the smallest margin between the first and second most likely predictions. The active machine learning approach improves the generalization and robustness of learning systems by selecting the subset of examples to learn from based on an informativeness criterion. Our aim is to learn the underlying concept with as few informative instances as possible. We highlight that our aim is not to compete with the state-of-the-art on robustness, but rather propose a general-purpose technique that equips classifiers with significant levels of robustness and generalization.

**Fig. 1:**
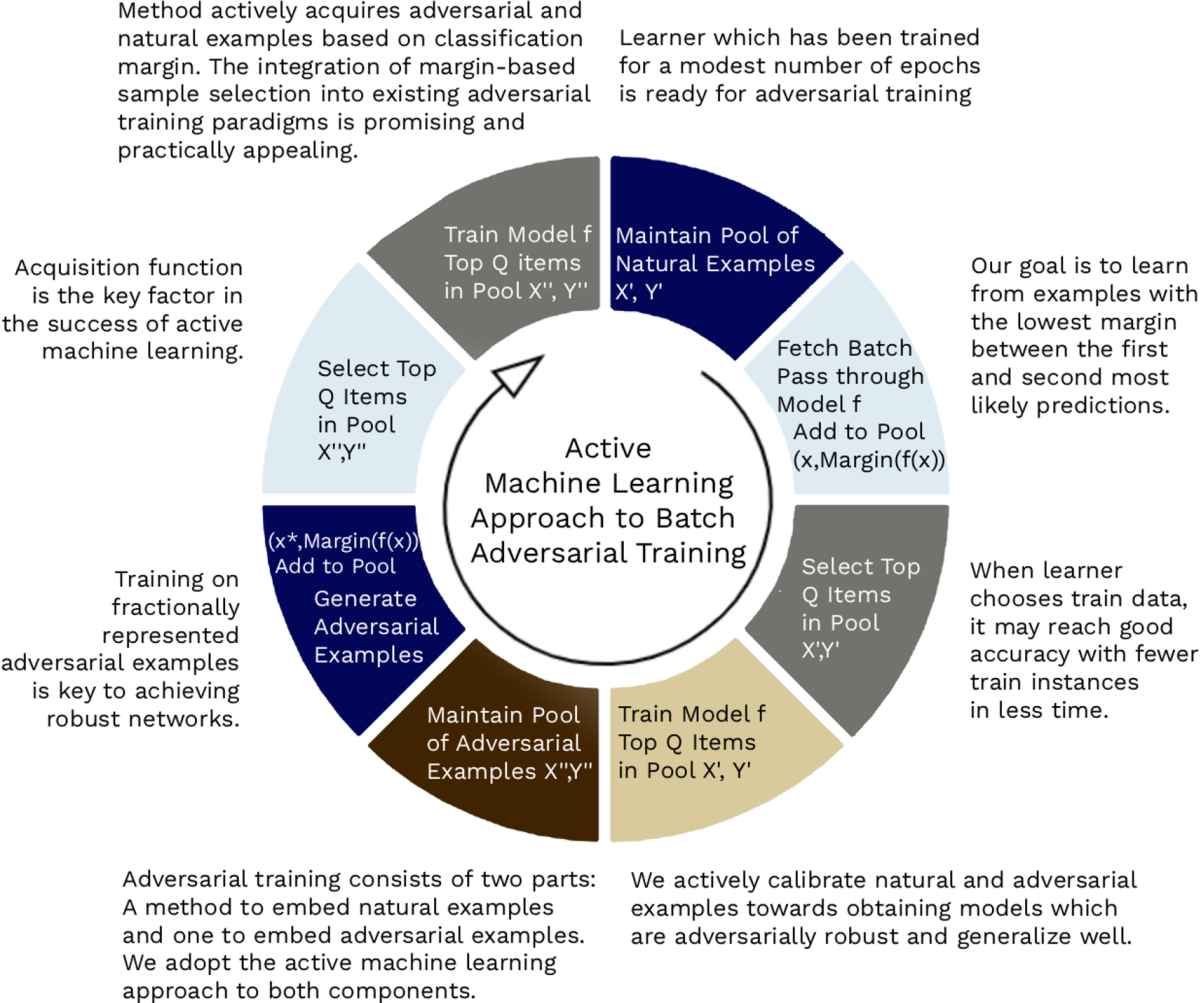
Overview of the active machine learning approach to adversarial training.

Among other defense strategies^19–24^, adversarial training is posed as an effective method to achieve robust and generalizable machine learning models^2,5,25,26^. Adversarial training seeks to solve a min-max optimization problem where the inner maximization searches for worst-case adversarial examples during batch training and the outer minimization optimizes across model parameters given natural and adversarial examples^27^. As training both robust and accurate learning systems remains an open problem, to enhance a model’s robustness and generalization capabilities during training is indeed, a significant endeavor. Our motivation is the training of deep neural networks which are generalizable and adversarially robust at the same time, thereby addressing key trade-off in adversarial training research (Fig 1).

As a whole, our contribution is a general method that confers significant levels of robustness and natural accuracy upon classifiers. Our work adopts the active machine learning approach to adversarial training where the key hypothesis is that if the learning algorithm chooses the training data from which it learns, it may reach good accuracy with less data in less time^11^. Active machine learning is powerful in situations where labeled data is expensive, therefore it is suitable to select more informative instances for labeling based on classification confidence, prediction variance, margin, entropy, or other informativeness criteria. Overall, we make the following contributions:

- We propose a novel data generation framework for adversarial training that incorporates active machine learning for the acquisition of adversarial examples. The method continuously learns from adversarial examples based on classification margin criterion. The general-purpose technique endows a classifier with significant levels of robustness and natural accuracy. We implement the active machine learning approach to adversarial training with multiple adversarial training methods such as Standard Adversarial Training (AT)^5^, Misclassification Aware adveRsarial Training (MART)^28^, and Theoretically Principled Trade-off between Accuracy and Robustness (TRADES)^6^. The method is highly modular and integratable into other adversarial training methods. This design makes the method straightforward to extend to new settings where different operations may be desired. This Article provides insights for future research on the accuracy-robustness trade-off with adversarial training.
- We perform a comprehensive battery of experiments to demonstrate the efficacy of the method, which makes it applicable for real-world applications (image recognition systems, autonomonous vehicles, computer and communications security, healthcare monitoring, etc.) where robustness and natural accuracy are of the essence.
- We provide theoretical contribution that sheds light on the properties of the active learning approach to adversarial training (Table 1).

**Table 1:** Overview of the characteristics of prominent adversarial training methods.

Method	Description	Advantages	Disadvantages
AT^5^	AT Trains on a mixture of natural and adversarial examples: Min-max optimization problem where the learning algorithm seeks to minimize loss and adversary seeks to maximize loss. This can be interpreted as learning to play an adversarial game with upper bound on the expected cost over adversarial samples^2^.	+ No decision making with individual instances + Computational efficiency	- Degradation in natural accuracy
MART^28^	Given that misclassified examples have a significant impact on the final robustness, MART introduces regularized adversarial risk which incorporates explicit differentiation of misclassified examples as a regularizer^28^.	+ Further improves robustness	- Computational complexity
TRADES^6^	Decomposition of prediction error for adversarial examples as the sum of natural classification error and boundary error. This decomposition provides a differentiable upper bound^6^. Regularization parameter introduced to be within range between 1 and 10 affects generalization and robust accuracy.	+ Seeks to balance robustness and generalization accuracy	- Trade-off between robustness and accuracy
SAT^29^	Given that ReLU activation function significantly weakens adversarial training due to its non-smooth nature, SAT replaces ReLU with its smooth approximations to strengthen adversarial training.	+ Improvement of adversarial robustness with no drop in accuracy & no increase in computational cost	- Requires architectural change
FAT^30^	Rather than employing most adversarial data maximizing the loss, FAT searches for least adversarial data minimizing the loss, among the adversarial data that are confidently misclassified.	+ Easy to implement by stopping PGD early + Computational efficiency	- Decrease in robustness to strong adversary
Generalist^31^	Generalist decouples natural generalization and robust generalization from joint training. It simultaneously trains base learners with task-aware strategies, combines them into a global learner.	+ Improvement in accuracy + Improvement in robustness	- Requires different learners -Requires different training strategies
HAT^32^	AT increases the margin along certain adversarial directions. HAT reduces this effect by incorporating additional wrongly labelled examples.	+ Improvement in accuracy + No degradation in robustness	- Introduction of more adversarial examples
SWA^33^	SWA performs simple averaging of multiple points along the trajectory of Stochastic Gradient Descent	+ Improvement in accuracy + Easy to implement + No computational overhead	- Requires change in training process
IAAT^34^	IAAT enforces sample specific perturbation margins around every training sample.	+ Improvement in test accuracy on unperturbed samples	- Marginal drop in robustness
GAIRAT^35^	GAIRAT assigns weights based on how difficult it is to attack a natural data point.	+ Improvement in accuracy + Improvement in robustness	- Computational complexity
CAT^36^	CAT develops a curriculum of adversarial examples generated by attacks with a wide range of strengths.	+ Improves generalization + Mitigates catastrophic forgetting	- Computational complexity - Requires the development of a curriculum of adversarial examples

This work directly addresses the key trade-off in adversarial robustness which remains a fundamental challenge in the field. This Article contributes to the broader scientific literature on adversarial training, generalization theory, and robust machine learning. The work acknowledges the resonance of the adversarial training process to the natural phenomenon known as the fractional quantum Hall effect^1,37^ in classical electrodynamics where fractional charge can change the characteristics of particles drastically. In what follows, we present the results of the proposed method which achieves significant improvements in accuracy and robustness with WRN34-10 and ResNet-18 architectures on CIFAR-10, CIFAR-100, SVHN, and TinyImagenet-200 datasets. Benchmark incorporates performance of standard Adversarial Training (AT), Misclassification-Aware adveRsarial Training (MART), and Theoretically Principled Trade-off between Robustness and Accuracy (TRADES). We subsequently present a detailed discussion and literature review on the accuracy-robustness trade-off of adversarial training which offers tremendous value to the research community in the area of robust machine learning. Finally, we present the method.

## Results

Through a comprehensive battery of experiments, we demonstrate that the active learning approach to adversarial training leads to significant improvements in robust and natural accuracy under various strong PGD^5^ adversaries. Because the proposed method can be integrated with adversarial training frameworks with few modifications, effective adversarial training methods can be special cases. We design our method for standard Adversarial Training (AT)^5^, Misclassification-Aware Adversarial Training (MART)^28^, and TRADES^6^.

We underscore the importance of proper pairing between deep neural networks and datasets as it pertains to adversarial robustness. We observed that the modest size of ResNet18 network can be considered a crucial factor when paired with a relatively large dataset such as CIFAR-100. This is supported through the contrasting of the (ResNet18, CIFAR-100) pair with (WRN34-10, CIFAR-10). More to the point, pairing a modest size network with a modest size dataset or a large network with a large dataset is likely to yield more robust networks upon adversarial training.

Our observation is that a smaller batch size results in more robust networks compared to a larger batch size. Our conjecture is that the current state of the network is paramount during the first step of projected gradient descent (perform an unconstrained gradient descent step). A large batch means that the network is not provided with the most recent optimization error. Moreover, the proposed method is subject to computational overhead compared to standard adversarial training which is introduced by the active selection step. This overhead is proportional to batch size. We find that a smaller batch size is likely to result in more robust networks.

### Threat model

The most basic adversary is one who does not utilize any projection function while attacking. A more advanced adversary is one who knows that the defender is utilizing a projection function but does not have inference time access to the defender and needs to conjecture the projection function while attacking. The most powerful adversary is one who can access not only the entire network parameters but also the defender’s system and projection function at any given time during inference. White-box attacks based on first-order optimization^5,38^ are considered state-of-the-art. We evaluate our framework with FGSM^2^, PGD-10^5^ and Auto-Attack (AA)^39^. AA is an ensemble of diverse attacks: APGD, APGD-DLR^39^, SQUARE^40^, and FAB^41^.

### Hyperparameters

Following best practice^42,43^, pre-training is performed for 10 epochs as initialization prior to adversarial training. We run the methods for 100 epochs for 2 rounds. We set $\epsilon = 8/255$ and $\alpha = 2/255$ for all adversaries alike. $\ell _\infty$-PGD-10 adversary is initialized with random start. We set the learning rate to 0.01 during training with batch size 128. With TRADES, we assign regularization parameter value 10 for balance between generalization and robustness.

### Datasets and networks

We evaluate the effectiveness of the data generation framework on benchmark datasets CIFAR-10, CIFAR-100^44^, SVHN^45^, and Tiny-Imagenet-200. CIFAR-10 consists of 50,000 training and 10,000 test instances across 10 classes. CIFAR-100 comprises 60,000 images within 100 classes. SVHN contains 73,257 training and 26,032 test instances across 10 classes. Tiny-Imagenet-200 contains 100000 images across 200 classes each class with 500 train instances and 100 test and validation instances. We use WRN34-10 and ResNet-18 architectures^46^ throughout the benchmarks.

### Metrics

The measure of accuracy^47^ shows how close overall model predictions are to the correct labels whereas the measure of robust accuracy shows how robust overall model predictions are in classifying adversarial examples. Robustness of the model is also measured by computing the attack success rate, i.e., the fraction of successful attacks on correctly classified test instances. This means that a lower success rate indicates higher robustness of the model. Furthermore, we adopt the recently proposed metric^48^ specifically designed as a balanced measure of robust and natural accuracy. For a balanced metric, F1-robust^48^ is the harmonic mean between robustness and natural accuracy. F1-robust is a powerful measure of the accuracy-robustness trade-off with adversarial training.

### Computational efficiency

The method generates full batches of adversarial examples and selects a subset of each batch for training. This incurs the computational cost of PGD for all samples within a batch. With the active machine learning approach, the learner reaches faster convergence as the learner chooses the data from which it learns. Therefore, faster convergence contributes more to the computational efficiency of the proposed method than reduced effective batch size. Table 3 compares training time of the method with baseline methods. Average excess compute time across datasets and networks with AT, MART, and TRADES is 3.33%, 8.24%, and 37.51%, respectively.

### Performance stability analysis

**Table 2:** Accuracy (%) and robustness (%) of training methods: Summary statistics, that is, mean performance across networks and datasets. We report the averaged results and confidence intervals. The performance improvements are reported in bold and italic. Our method confers significant levels of robustness and accuracy upon training methods.

Method	Accuracy	Robust accuracy		
		FGSM	PGD	AA
Standard training	60.72 ± 0.53	4.81 ± 0.36	0.65 ± 0.14	0.4 ± 0.10
AT	53.54 ± 0.43	30.89 ± 0.60	26.44 ± 0.43	24.68 ± 0.41
MART	52.41 ± 0.77	33.06 ± 0.64	29.84 ± 0.51	24.70 ± 0.47
TRADES	50.62 ± 0.90	30.88 ± 1.55	27.77 ± 0.88	23.91 ± 0.69
Ours (AT)	56.23 ± 0.42 ***(+2.69)***	33.51 ± 0.73 ***(+2.62)***	28.30 ± 0.45 ***(+1.86)***	26.36 ± 0.44 ***(+1.68)***
Ours (MART)	56.31 ± 1.22 ***(+3.9)***	35.42 ± 0.78 ***(+2.36)***	31.13 ± 0.62 ***(+1.29)***	26.40 ± 0.60 ***(+1.7)***
Ours (TRADES)	55.79 ± 1.22 ***(+5.17)***	32.55 ± 1.10 ***(+1.67)***	29.07 ± 0.81 ***(+1.3)***	24.57 ± 0.72 ***(+0.66)***

**Fig. 2:**
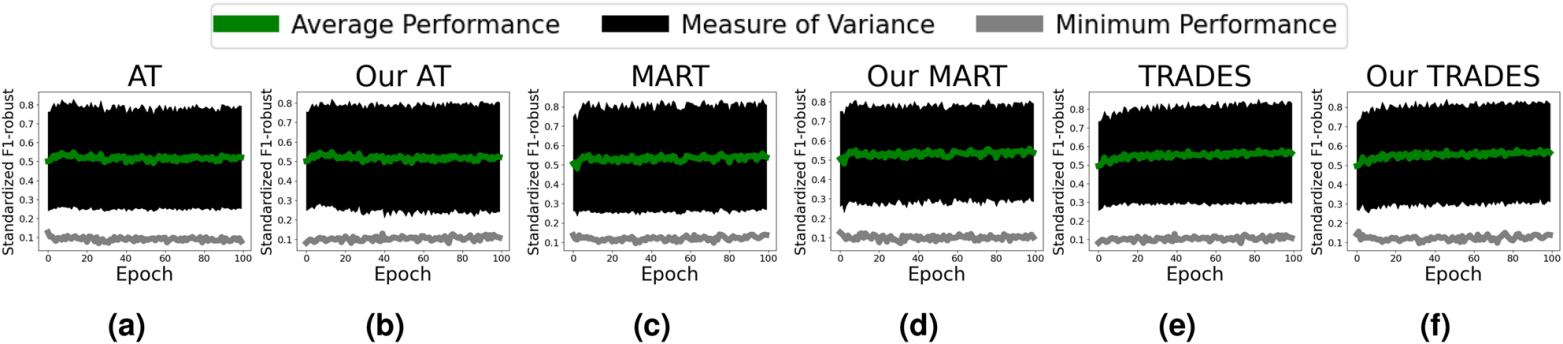
Stability over time across runs.

Selection mechanism is based on the current model’s margin which depends on training dynamics such as different training trajectories. This suggests that training trajectory could significantly vary depending on initialization or randomness. The coupling of model-dependent selection with adversarial training requires examination of stability across runs over early/late training stages. Here, we report results validates consistently robust method. Figure 2 depicts variance in performance outcomes across runs. We observe similar trends, spikes, and variance ranges between adversarial training methods and the active machine learning approach to the methods. Furthermore, we observe relative stability over time during early and late stages of training.

### Overview

**Table 3:** F1-robust (%) and computation time (hours) of training methods on CIFAR-10 and SVHN. F1-robust is the harmonic mean between robustness and natural accuracy. The performance improvements are reported in bold and italic. The additional computation time is reported in bold. Our method consistently improves F1-robust of adversarial training methods. This finding is significant in addressing the accuracy-robustness trade-off with adversarial training.

Method	F1-robust			Compute
	FGSM	PGD	AA	
Standard training	9.93 ± 0.54	1.68 ± 0.24	1.04 ± 0.18	10.82 ± 0.68
AT	40.55 ± 0.57	35.94 ± 0.46	34.12 ± 0.45	43.35 ± 6.47
MART	42.10 ± 0.70	38.86 ± 0.62	33.79 ± 0.60	44.44 ± 18.36
TRADES	39.5 ± 1.4	34.91 ± 1.55	31.10 ± 0.88	27.06 ± 0.69
Ours (AT)	43.83 ± 0.60 ***(+3.28)***	38.6 ± 0.44 ***(+2.66)***	36.65 ± 0.44 ***(+2.53)***	44.8 ± 4.35 ***(+1.45)***
Ours (MART)	45.37 ± 0.91 ***(+3.27)***	41.21 ± 0.81 ***(+2.35)***	36.46 ± 0.80 ***(+2.67)***	46.97 ± 3.10 ***(+2.53)***
Ours (TRADES)	42.9 ± 1.18 ***(+3.4)***	39.22 ± 0.99 ***(+4.31)***	34.8 ± 0.92 ***(+3.7)***	51.97 ± 42.79 ***(+24.91)***

**Fig. 3:**
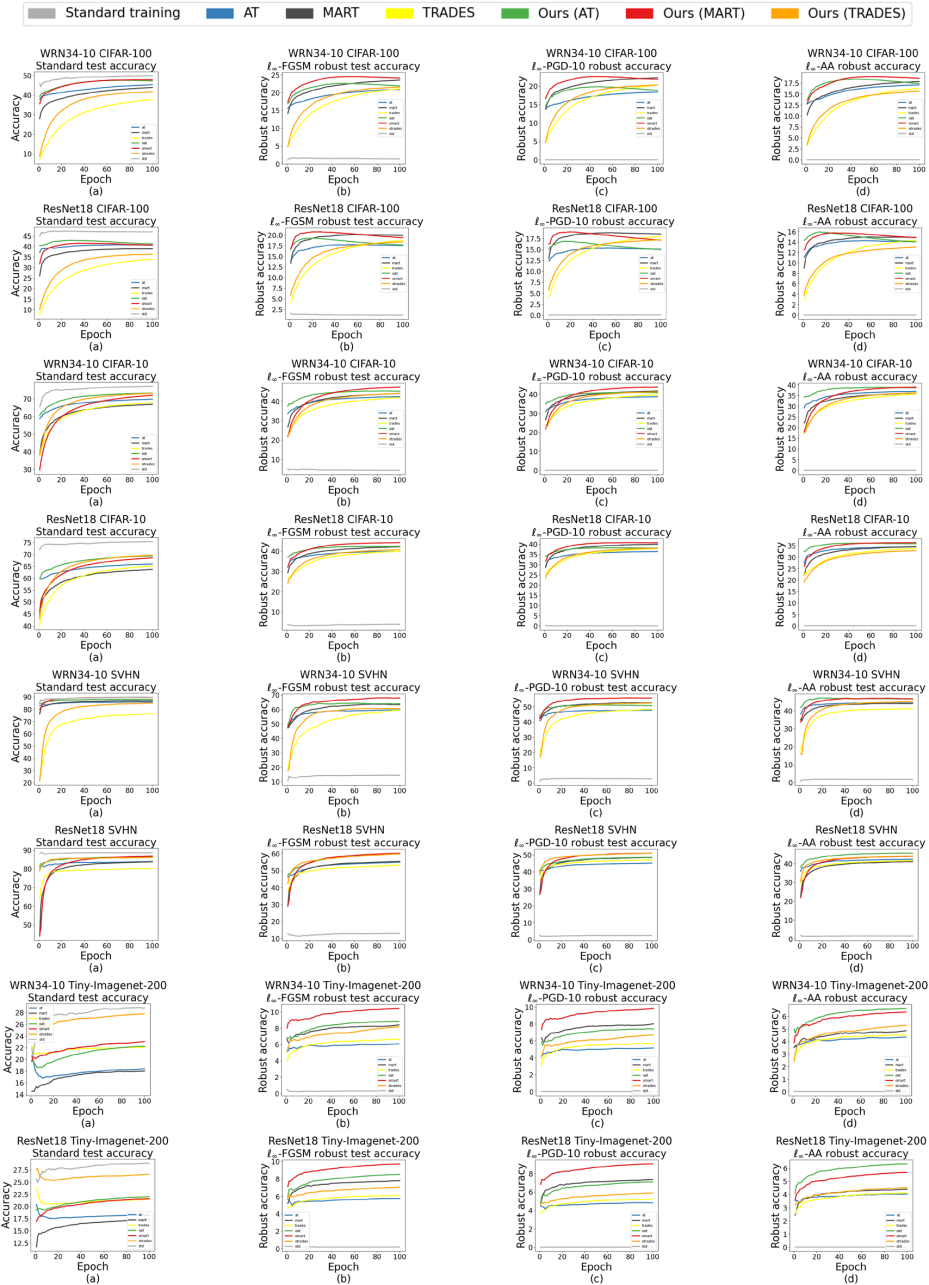
The measure of standard test accuracy shows how close overall model predictions are to the correct labels at test time. Robust test accuracy shows how effective overall model predictions are in classifying adversarial examples at test time. We observe reasonable improvement through our data generation framework with natural accuracy and robust accuracy.

Summary performance statistics from Tables 2 and 4 suggest the active machine learning approach to adversarial training is able to significantly improve average and best performance checkpoints with adversarial training methods. Analysis of results from Table 2 reveals insights into the efficacy of the method which significantly improves natural accuracy and robustness against $\ell _\infty$-FGSM, $\ell _\infty$-PGD, and $\ell _\infty$-AA adversaries compared to AT, MART, and TRADES on CIFAR-10, CIFAR-100 and SVHN datasets. With the goal of gaining deeper insights into the performance of the method, we visualize average plots (Fig. 3) which show reasonable improvement through the data generation framework with natural accuracy and robust accuracy. For smoother visualization, we time-average the performance for each method through cumulative moving average.

**Fig. 4:**
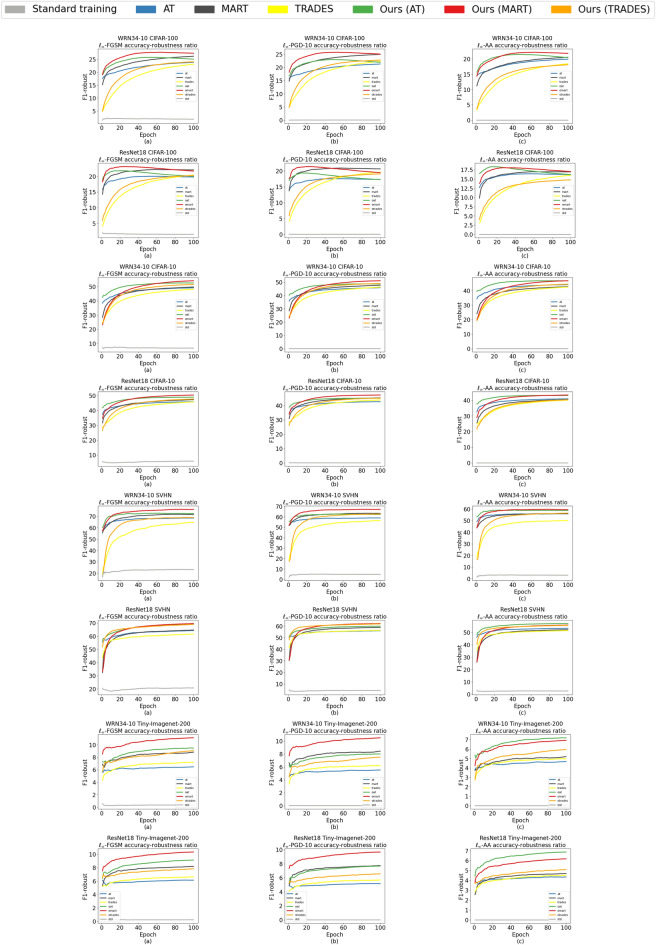
F1-robust, introduced as a harmonic mean of standard test accuracy and robust test accuracy, indicates the accuracy-robustness trade-off with adversarial training. We demonstrate F1-robust for $\ell _{\infty }$-FGSM, $\ell _{\infty }$-PGD and $\ell _{\infty }$-AA adversaries with ResNet18 and WRN34 on CIFAR-10, CIFAR-100, SVHN, and Tiny-Imagenet-200 datasets.

Method’s senior performance prompted us to perform complimentary analysis of the accuracy-robustness trade-off through F1-robust, introduced as a harmonic mean of standard test accuracy and robust test accuracy. Results from Table 3 show that the data generation framework significantly improves F1-robust. This finding is significant in addressing the accuracy-robustness trade-off with adversarial training. This is also supported by Fig. 4 which indicates that the data generation method confers a higher accuracy-robustness ratio compared to AT, MART, and TRADES. This quality makes the data generation method suitable for areas where robustness and natural accuracy are of the utmost importance.

In the following, we further investigate the details of performance gains in natural and robust accuracy achieved through the proposed method as well as introduced computational overhead. We investigate AT, MART, and TRADES from 4 different perspectives: (1) natural accuracy (2) robust accuracy against FGSM adversary (3) robust accuracy against PGD-10 adversary (4) robust accuracy against powerful ensemble Auto-Attack adversary. Next, we discuss the accuracy-robustness trade-off of adversarial training methods as measured by F1-robust. Furthermore, we compare performance metrics considering best checkpoints and average statistics to examine the generalizability of the method as well as provide ablation on the paramater Q in Algorithm 1 which decides the number of examples to train on at each batch during training.

### Natural accuracy

Firstly, we review the method in terms of the measure of natural accuracy. Natural accuracy obtained through the proposed method for AT, MART, and TRADES is consistently and significantly superior to that obtained through these adversarial training frameworks. This finding is associated with summary statistics in Table 2 where the active machine learning approach has improved the generalization accuracy of all adversarial training methods up to 5.09%. Moreover, Table 6 under accuracy shows the method has improved, for instance, the generalization of TRADES 5.88% with ResNet18 and SVHN. This pattern of improvement in generalization is indeed, outstanding to the challenge of achieving through adversarial training robust networks which are accurate.

### FGSM adversary

Secondly, we analyze the performance of the proposed method under FGSM adversary which is a simple and fast technique of generating adversarial examples. Summary performance statistics from Tables 2 and 4 suggest the active machine learning approach to adversarial training is able to substantially improve average and best performance checkpoints of FGSM robustness with all adversarial training methods. Proper pairing of the dimensions of network architectures and datasets is key to achieving robust networks through adversarial training. In Table 6, we discern a valuable insight in the less than 1% decrease in robust accuracy with MART and TRADES under ResNet18 architecture and CIFAR-100 dataset. We speculate that the modest size of ResNet18 network can be considered an important factor in this observation. This learning is evident when contrasted with the increase in robust accuracy under the same dataset and adversarial training frameworks with the larger WRN34-10 network.

### PGD adversary

Thirdly, we investigate PGD-10 adversary which is considered state-of-the-art in the literature. Summary performance statistics from Tables 2 and 4 suggest the method significantly improves PGD robustness up to 1.73% on average and 3.53% with the best checkpoint. According to Table 6 under PGD robust accuracy, we discern up to 4.03% enhancement of robust accuracy with ResNet18 and SVHN. Results from other adversarial training frameworks also exhibit noticeable improvement in robustness against PGD adversary. Above-mentioned best practice of pairing network architectures and datasets of suitable dimensions resonates with PGD adversary alike where the pairing of ResNet18 and CIFAR-100 (a modest size network and large dataset) resulted in a decrease in robust accuracy under the proposed data generation framework compared to AT, MART, and TRADES. The method notably improves F1-robust measure of accuracy-robustness trade-off of MART with WRN34-10 and CIFAR-100 in exchange for the less than 0.5% decrease in robust accuracy under Table 7. Over and above that, we observe significant and consistent improvement in robust accuracy under PGD adversary and WRN34-10 architecture with all methods and datasets indicating the efficacy of the active machine learning approach to adversarial training.

### AA adversary

Next, we inspect robust accuracy under strong ensemble AA adversary which is considered a powerful threat model in the adversarial machine learning literature. Summary performance statistics from Tables 2 and 4 suggest the method is able to significantly improve average and best performance checkpoints of AA robustness up to 1.8% on average and 1.5% with the best checkpoint. Robust accuracy of AT, MART, and TRADES, according to Table 6, improved under AA adversary with ResNet18 and SVHN as well as under WRN34-10 with CIFAR-10 and SVHN. We observe a decrease in robust accuracy under the method with TRADES and CIFAR-100 which we attribute to the dataset-architecture pairing as well as the fine-tuning of TRADES’s regularization hyperparameter as earlier. We believe that the selection of the sweet spot between robustness and accuracy as regularized by this parameter is key in achieving robust networks through TRADES which can only be identified through further experimentation. Over and beyond that, the active machine learning approach to adversarial training also enhanced the robustness performance of AT and MART with ResNet18 and CIFAR-10 as well as with WRN34-10 and CIFAR-100.

### F1-robust

Ultimately, we evaluate F1-robust measure of accuracy-robustness trade-off under Table 3 which demonstrates that the active machine learning approach to adversarial training prominently upgrades the accuracy-robustness trade-off. This improvement of the accuracy-robustness trade-off achieved through the proposed data generation framework is significant in addressing the accuracy-robustness trade-off of adversarial training. We observe consistent improvment of the accuracy-robustness trade-off of adversarial training methods AT, MART, and TRADES under FGSM, PGD, and AA adversaries with WRN34-10 architecture and CIFAR-10, CIFAR-100, and SVHN datasets as well as with ResNet18 architecture and CIFAR-10 and SVHN datasets. We find the best practice of the proper pairing of network architectures and datasets all the more relevant pertaining to the accuracy-robustness trade-off of MART and TRADES under (ResNet18, CIFAR-100) pairing as contrasted with (WRN34-10, CIFAR-100) pairing.

### Robust overfitting

Robust overfitting^49^ is considered a general phenomenon for adversarially robust deep learning. This phenomenon is of prominence when contrasted with standard training of deep neural networks where it is common practice to train for as long as possible to minimize training loss. In the case of adversarial training, however, it is empirically demonstrated that there is a gap between the best robust test performance during adversarial training and the final robust test performance at the end of adversarial training. Therefore, it is important to compare best robustness checkpoints with average robustness measures in awareness of the robust overfitting phenomenon which encourages one to stop adversarial training early to achieve the best robustness. We provide the best checkpoints in Tables 4 and 8 to clarify whether the observed improvements over the baselines hold when considering the best checkpoint results addressing the concern of robust overfitting.

**Table 4:** We provide best checkpoints to address the concern of robust overfitting.

Method	FGSM		PGD		AA	
	Best	Last	Best	Last	Best	Last
Standard training	8.22	5.42	1.64	0.7	0.98	0.45
AT	35.59	32.75	30.16	28.08	28.03	26.20
MART	38.07	34.26	34.09	30.53	28.68	25.75
TRADES	37.34	33.23	32.44	30.24	27.99	26.38
Ours (AT)	39.14 ***(+3.55)***	34.65	33.27 ***(+3.11)***	28.4	29.68 ***(+1.65)***	26.20
Ours (MART)	40.92 ***(+2.85)***	38.42	35.7 ***(+1.61)***	31.75	29.9 ***(+1.22)***	26.7
Ours (TRADES)	39.05 ***(+1.71)***	35.29	33.37 ***(+0.93)***	30.69	28.42 ***(+0.43)***	26.35

### Ablation

The parameter Q in Algorithm 1 controls the number of selected samples per batch. Q significantly affects the behavior of the model. Here, we provide ablation which shows the impact of different Q values on natural accuracy, robust accuracy, and convergence speed with ResNet18 on CIFAR-10. Summary statistics in Table 5 demonstrate the impact of the parameter Q on accuracy and robustness where a larger Q results in better generalization accuracy and robustness against FGSM, PGD, and AA adversaries. A larger Q also results in excess compute time.

**Table 5:** Ablation on the parameter Q. B is batch size. A larger Q yields more generalizable and robust networks at higher compute time.

Method	Q	Accuracy	FGSM	PGD	AA	Compute
Ours (AT)	$0.25\times B$	62.28 ± 3.07	40.23 ± 2.30	37.32 ± 2.14	36.20 ± 2.04	19.02
Ours (AT)	$0.50\times B$	69.36 ± 0.36	42.32 ± 0.21	38.05 ± 0.19	35.89 ± 0.18	18.94
Ours (AT)	$0.75\times B$	71.76 ± 2.04	42.38 ± 1.99	37.35 ± 2.16	35.10 ± 2.17	19.96
Ours (MART)	$0.25\times B$	58.35 ± 4.21	38.87 ± 3.29	36.75 ± 3.09	32.97 ± 3.16	16.5
Ours (MART)	$0.50\times B$	68.60 ± 0.64	44.22 ± 0.35	40.73 ± 0.28	36.37 ± 0.30	19.12
Ours (MART)	$0.75\times B$	71.45 ± 3.73	45.50 ± 2.86	41.17 ± 2.52	36.33 ± 2.60	17.45
Ours (TRADES)	$0.25\times B$	60.58 ± 6.82	36.23 ± 5.37	34.61 ± 5.01	28.96 ± 4.45	19.55
Ours (TRADES)	$0.50\times B$	69.66 ± 0.68	40.75 ± 0.51	37.97 ± 0.45	32.88 ± 0.47	18.91
Ours (TRADES)	$0.75\times B$	68.06 ± 5.20	43.34 ± 4.52	40.74 ± 4.08	36.03 ± 4.26	19.37
Summary Statistics	$0.25\times B$	60.4 ± 5.20	38.44 ± 3.61	36.22 ± 3.16	32.71 ± 4.01	18.35
	$0.50\times B$	69.20 ± 0.68	42.43 ± 0.51	38.91 ± 0.45	35.04 ± 0.47	18.99
	$0.75\times B$	70.42 ± 4.21	43.74 ± 3.10	39.75 ± 3.12	35.82 ± 2.69	18.92

## Discussion

The gain of robustness is usually at the expense of natural accuracy. For this reason, we proposed the active machine learning approach to adversarial training which achieves significant levels of robustness and generalization by selecting the subset of examples to learn from based on an informativeness criterion. We hope that it will help real-world applications achieve robust models. Having said that, the method requires additional computation compared to standard adversarial training.

Our work focuses on the acquisition of adversarial examples based on classification margin criterion. In the future, we may incorporate classification uncertainty and entropy as informativeness criteria in the context of the active machine learning approach to adversarial training. It is important to note, however, the incorporation of other informativeness criteria requires close examination of the specific characteristics of each informativeness measure such as whether adversarial training must include the hardest examples or a sliding window apparatus throughout training in order to achieve the best generalization and robustness results. The comparison of margin with alternative informativeness measures will provide further insights into the proposed approach.

The work utilizes adversarial examples to address the accuracy-robustness trade-off with adversarial training. In the future, we may harness counterfactual examples to improve the interpretability and explainability of learning systems. More specifically, projected gradient descent unveils compelling truths about the presence of superposition, adversarial robustness, and in turn, mechanistic interpretablity. Measuring interpretability through feature superposition allows to select more interpretable learning trajectories at train time as well as provide explainable references at inference time. This is significant to the challenge of interpreting the circuitry inside deep neural networks. This research direction is of broader interest as it paves the way for training more interpretable networks. In conclusion, we encourage to optimize for generalization, adversarial robustness, and interpretability.

## Methods

Given an instance-label pair $(x,y)$ from an unknown data distribution $p$ and a classifier $f$, an adversarial example is a perturbed instance $x^*=x+\delta$ which is misclassified by the model, i.e. $f(x^*)\ne y$. Here, we concentrate on the Fast Gradient Sign Method^2^ and the $\ell _\infty$-white-box attack^5^ that maximizes the training loss using projected gradient descent. Training on adversarial examples during training is an established defense^50–56^. Other commonly used methods for defending deep learning models from adversarial examples are Network Distillation^19^, Adversarial Detecting^57^, Input Reconstruction^58^, Classifier Robustifying^59^, Network Verification^60^, and an ensemble of them^61,62^. Adversarial training^3^ aims to minimize the worst-case error when the data is perturbed by adversary^2^. In adversarial training, the learning algorithm seeks to estimate parameters for the model that classify instances correctly whereas adversary seeks to choose examples that will cause the model to choose other classes. Let $(X,d_\infty )$ be the input feature space $X$ with the infinity distance metric $d_\infty (x,x^\prime ) = \Vert x - x^\prime \Vert _\infty$ and

$$\begin{aligned} B_\epsilon [x] = \{x^\prime \in X | d_\infty (x,x^\prime ) \le \epsilon \} \end{aligned}$$

be the closed ball of radius $\epsilon> 0$ centered at $x$ in $X$^30^. Given a dataset $S = \{(x_i, y_i)\}_{i=1}^n$, where $x_i \in X$ and $y_i \in Y = \{0,1,...,C - 1\}$, the objective function of adversarial training^5^ is

$$\begin{aligned} \min _{f \in F}\frac{1}{n}\sum _{i=1}^n\{\max _{\bar{x} \in B_{\epsilon }[x_i]}l(f(\bar{x}),y_i)\} \end{aligned}$$

where $x^*$ is the adversarial data within the $\epsilon$-ball centered at $x$, $f(.) : X \rightarrow \mathbb {R}^C$ is a score function, and the loss function $l : \mathbb {R}^C \times Y \rightarrow \mathbb {R}$ is a composition of a base loss $l_B : \Delta ^{C-1} \times Y \rightarrow \mathbb {R}$ and an inverse link function $l_L : \mathbb {R}^C \rightarrow \Delta ^{C-1}$ in which $\Delta ^{C-1}$ is the corresponding probability simplex^30^.

Adversarial training is different from data augmentation methods. Data augmentation is an effective method of improving the generalization capability of machine learning models, especially with datasets that exhibit class imbalance or lack of quality. The main goal of data augmentation is to improve the accuracy of learning models so that they generalize well to unseen data points. Moreover, effective data augmentation strategies reduce complexity and enable simpler models to achieve good generalization^63^. In general, data augmentation is performed by either transforming training samples in a desired manner or creating new samples^64^. Augmentation usually incorporates transformations that are expected to occur in the test set whereas adversarial examples are unlikely to occur naturally^2^. The goal of adversarial training is to achieve adversarial robustness, i.e. to improve the model’s performance on test data with adversarial perturbations. In adversarial training, generated adversarial examples are usually annotated with the source class label^27^ so that a robust classifier correctly labels adversarial examples.

While the Fast Gradient Sign Method^2^ and the $\ell _\infty$-white-box attack^5^ are effective adversarial perturbation methods utilized within adversarial training frameworks, different machine learning paradigms may require different adversarial perturbation methods. Multi-Instance Learning (MIL), for example, is a weakly supervised machine learning paradigm where information is available for a set of instances referred to as bags and not for every instance. MIL describes each sample as a bag of many instances and supervised information is provided at the bag level. Effective adversarial perturbation methods Multi-Instance Customized Adversarial Perturbation (MI-CAP) and Multi-Instance Universal Adversarial Perturbation (MI-UAP)^65^ have been proposed to provide suitable adversarial perturbations in the MIL context. As the number of instances in each bag is not uniform and gradients which correspond to different bags typically exhibit varying shapes, MI-CAP and MI-UAP average each row and utilize attention-based MIL methods.

Standard Adversarial Training (AT)^2,3^ seeks to minimize the cross entropy loss on adversarial examples with respect to original class labels. Adversarial training has been enhanced in numerous ways^26^. An adversarial training method that enhances robustness while also improving accuracy is smooth adversarial training^29^. This method replaces the ReLU activation function with smooth approximations to strengthen adversarial training. This study^30^ proposes a novel formulation of Friendly Adversarial Training (FAT) where they search for the least adversarial data minimizing the loss rather than employing the most adversarial data maximizing the loss among the adversarial data that are confidently misclassified. Considering the individual characteristics of adversarial robustness, Instance Adaptive Adversarial Training (IAAT)^34^ aims to perform adversarial training at the instance level where $\epsilon$ is selected to be as large as possible, ensuring images within the $\epsilon$-ball of *x* are from the same class. Adversarial Defense via Surrogate-loss minimization (TRADES)^6^ uses the Kullback-Leibler (KL) divergence as a regularization term to push the decision boundary away from the data. Motivated by the finding that misclassified examples have a significant impact on robustness, Misclassification Aware Adversarial Training (MART) explicitly differentiates misclassified and correctly classified examples during training^28^. Curriculum Adversarial Training (CAT)^36^ develops a curriculum of adversarial examples generated by attacks with a wide range of strengths to mitigate the forgetting and the generalization issues. CAT yields a performance on non-adversarial inputs that is comparable to the state-of-the-art models. Helper-based Adversarial Training (HAT)^32^ reduces the effect of unwarranted increase in the margin along certain adversarial directions by incorporating additional wrongly labelled examples during training. Stochastic Weight Averaging (SWA)^33^ averages multiple points along the trajectory of SGD to flatten the adversarial loss landscape^66,67^ which leads to better generalization than standard training. Universal Inverse Adversarial Training (UIAT) encourages the model to produce similar output probabilities for an adversarial example and its inverse adversarial counterpart^68^. A recent study presents LBGAT^69^ that incorporates Mean Square Error (MSE) regularization term between the logits of the natural model, alongside the robust one. Generalist^31^ is composed of two base learners trained within their respective domains and a global learner that aggregates the parameters of base learners during the training process. A recent study^70^ introduces adversarial examples into the active learning pipeline in order to expand the labeled dataset. While existing research^70^ indicates that adversarial training may be incorporated into the active learning pipeline, the harnessing of active learning for the acquisition of adversarial examples is novel and powerful.

Many works have studied several aspects of the adversarial training process such as using different loss functions^6,32,71^, tuning learning rate or weight decay parameters, adjusting the strength of the attack at training time, and incorporating label smoothing and stochastic weight averaging^66,72^. Existing weighted approaches simply assign weights to adversarial examples. GAIRAT^35^, for example, assigns weights to adversarial examples based on how close or far they are from the class boundary given how difficult it is to produce adversarial examples from natural inputs. Moreover, MART^28^ assigns different weights to correctly classified and misclassified adversarial examples. Our approach simply selects adversarial examples with low classification margin between the first and second most likely predictions. Our approach is not a weighted one but rather one of active selection.

In this Article, we propose a novel data generation framework for adversarial training that is computationally efficient and adheres to the spirit of active machine learning. In general, the method can be applied to adversarial training frameworks. Adversarial training consists of two parts: a method to embed natural examples and one to embed adversarial examples. Here we adopt the active machine learning approach to both components. Algorithm 1 summarizes our data generation approach to adversarial training. This research provides insights for future research on the accuracy-robustness trade-off of adversarial training.

**Algorithm 1:**
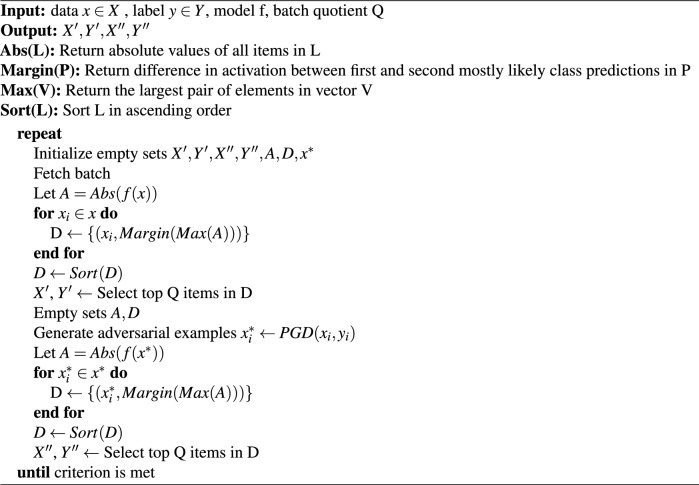
Data generation Algorithm.

Recent research considers multiple distance metrics such as $L_p$ norm of $x-x^*$ for $p=0$^38^, $p=1$^73^, $p=2$^74^, and $p=\infty$^2,5^. In our work, we follow^5^ to consider $d(x,x\prime )=\ell _\infty (x-x^*)$. We emphasize that our approach can be adapted to other distance metrics. Loss objective is the cross entropy loss between input logits and target in the case of standard adversarial training and Kullback-Leibler divergence loss in the case of TRADES and MART. We use capital letters to represent sets of vectors. Symbols X’ and Y’ in Algorithm 1 store natural examples whereas X” and Y” store adversarial examples. $x_i \in X$ refers to one instance from the batch.

We emphasize that the number of adversarial examples introduced in the training phase is the same as in standard adversarial training. We clarify that $PGD(x_i,y_i)$ generates one adversarial example per training sample. Selection of examples is performed globally across all classes. Moreover, Q is the number of examples to train on at each batch. The same Q applies to both natural and adversarial examples. The impact of the parameter Q in Algorithm 1 is on the phenomenon of overfitting to the training set as well as computational complexity. A higher Q may result in overfitting as well as more compute time during adversarial training while a lower Q may result in lower degrees of robustness and accuracy. We find that the choice of Q is case-specific and must be parameterized according to to the specific model and data. The method treats each batch as is without considering class imbalance.

The method selects samples with the smallest margin, that is, the most uncertain ones. Focusing on the hardest samples improves both generalization and robustness. This is because the goal of standard training and adversarial training processes is to estimate the true manifolds. Training on fractionally represented instances, that is, instances that seemingly belong to more than one class at the same time, is key to estimating the true manifolds and achieving both generalizable and robust models. In other words, the least certain a model is at train time about the exact apparatus of a fractionally represented instance, the more improvement in both generalization and robustness training on such an instance will yield. Although selecting the most uncertain instances during training may lead to overfitting to seemingly ambiguous instances at early stages of training, it will lead to more generalizable and robust models in the long run. More to the point, adversarial examples render useful in achieving not only robust but also generalizable models. In order to overview what are adversarial examples, let us begin with the fractional quantum Hall effect^37^ in physics: classical electrodynamics to be exact. Protons and electrons have the property of being excluded from being in the same quantum mechanical state, that is in the same position. Quantum numbers, however, also turn out to be fractional. This is because bits of magnetic field can get attached to each electron creating other objects. These composite particles have properties very different from those of electrons. Magnetic field drastically changes the characteristics of particles. Likewise, adversarial examples are in a state of superposition, that is both one and zero at the same time. A scholarly understanding of adversarial examples is to optimize input to maximize prediction error. The precise configuration of such an instance is a random artifact of the variability in different runs of backpropagation. Adversarial examples are result of superposition: neurons representing more features than there are dimensions. In the superposition hypothesis, model embeds more features than there are neurons^1^. Polysemanticity (neural networks packing many unrelated concepts into a single neuron) is inevitable when this happens. While polysemanticity helps store additional sparse features in superposition, it leads to less robust models. For polysemanticity to cause superposition is plausible because it is possible to have n orthogonal vectors in n-dimensional space and it is possible to have many more almost orthogonal vectors ($< \epsilon$cosine similarity) in high-dimensional space (Johnson-Lindenstrauss Lemma^75^). Sparsity also makes it possible to project a vector into lower-dimensional space and reconstruct it back to the original vector. Concretely in the superposition hypothesis, features are represented as almost orthogonal directions in the vector space of neuron outputs^1^. Superposition simply means there are more features than dimensions. With almost orthogonal features, one feature activating looks like other features slightly activating. Superposition can allow a model to represent extra features and the number of extra features increases as we increase sparsity^1^. Superposition happens when features have fractional dimensionality. Adversarial robustness sharply decreases as supersposition forms and the level of vulnerability to adversarial examples closely tracks the number of features per dimension^1^. Superposition can cause models to be vulnerable to adversarial examples. In other words, adversarial examples are result of superposition, that is, neurons representing more features than there are dimensions. Adversarial robustness is inversely correlated with the number of features stored in superposition per dimension. Superposition can explain why adversarial examples exist^1^. It can also justify why focusing on the hardest examples during training improves both generalizationn and robustness.

### Theoretical analysis

In this section, we discuss the problem of optimal allocation of resources in the conducting of experiments. We will bridge the gap between optimal experimental design, maximal expected information gain in the conducting of experiments, and the measuring of robust error as the sum of natural error and boundary error. We will clarify that adversarial examples that add the most amount of information as measured by an informativeness criterion such as $\Omega$ to the model’s concept during training contribute more to adversarial robustness than randomly selected adversarial examples.

The mathematical apparatus of experimental design addresses not deterministic but random quantities^10^. The effectiveness of applying experimental design methods depends on their appropriate utilization. Our aim is to develop an estimator of unknown parameters not depending on a concrete form of the distribution function of the observations. Experimental design allows us to effectively extract information about unknown parameters being estimated^10^. Let the loss in the case of an incorrect decision be characterized by the quantity $R=W_1*D+W_2*L$ where $W_1$ and $W_2$ are weight multipliers, *D* is the loss for an incorrect decision and *L* is the loss for not determining estimates of unknown parameters with sufficient accuracy^10^. This representation is a compromise between two contradictory requirements: *R* as a function of *D* and *L* must describe the real situation. If the probability of one of the models is close to one, then $W_1*D < W_2*L$. If the probabilities of the models are close to one another, then $W_1*D> W_2*L$. When none of the hypotheses is preferred, $W_1$ equals unity and $W_2$ equals zero and the problem reduces to the problem of discriminating hypotheses. When one of the hypotheses is known to be true, the problem reduces to the problem of seeking estimates of parameters.

We consider a sequential procedure for designing experiments. At each of the *N* stages, a point $X_N$ is sought that corresponds to $max(R(N)-R(N+1))$. The last observation must be allocated to the point $X_N$. For nonlinear parameterization, the representation of results in terms of estimates of unknown parameters is not always possible^10^. In such cases, it is natural to turn to the posteriori probability distribution function *p*(*x*) in the space of parameters. For the construction of the best linear estimates, it is sufficient to know only the first and second moments of the function. Therefore, the description of the results of an experiment using the posteriori probability distribution function *p*(*x*) requires prior information than the description of these results using the techniques of the best linear estimates. Since *p*(*x*) is defined on a multidimensional surface, the comparison of the results of various experiments directly from the form of *p*(*x*) is cumbersome. From a conceptual point of view^10^, it is necessary to address situations where it is difficult to state a preference for one or another experiment, relying directly on the form of *p*(*x*). For this reason, one may choose a measure of information such as classification margin or entropy which is of utility with the design of experiments for discriminating hypotheses. If the density of the distribution in the space of parameters is *p*(*x*), then classification margin is equal to $\min _{i}w^Tx_i y_i$ where $w^Tx_i$ is the prediction and $y_i$ is the ground truth and entropy (the measure of lack of order) is equal to $-\int p(x)\ln p(x) dx$^10^. Let an experiment be conducted with a set of observations. Then the measure of acquired knowledge will be a function of the posteriori density of the parameters. In experimental design, we rely on the expectation of the measure of the amount of of information gained in the experiment with *p*(*x*)^10^.

Adversarial training extends regular training to a case where there exit two optimization criteria: one for the learner and one for adversary. Robust error can be bounded by two terms^6^: One term corresponds to the natural error and the other term corresponds to how likely input features are close to the $\epsilon$-extension of the decision boundary known as the boundary error. In this way, robust error is decomposed as the sum of natural error and boundary error. Given a set of instances $x_1,...,x_n \in X$ and labels $y_1,...,y_n \in \{-1,+1\}$, let data be sampled from an unknown distribution $(X,Y) \sim D$. To characterize the robustness of a score function $f: X \rightarrow \boldsymbol{R}$^76^, defined robust error as $R_{Rob}(f):= \mathbb {E}_{(X,Y)\sim D} 1\{\exists X' \in \mathbb {B}(X,\epsilon ) s.t. f(X')Y\le 0\}$. Natural error is defined as $R_{Nat}(f):= \mathbb {E}_{(X,Y)\sim D} 1\{f(X)Y\le 0\}$. It is evident that $R_{Rob}(f) \ge R_{Nat}(f)$ for all *f*. Equality holds when $\epsilon =0$. Boundary error is defined as $R_{Bdy}(f):=\mathbb {E}_{(X,Y)\sim D} 1 \{X \in \textbf{B}(DB(f),\epsilon ), f(X)Y> 0 \}$. Therefore, we have $R_{Rob}(f) = R_{Nat}(f)+R_{Bdy}(f)$.

The expected increment of information for any design is nonnegative^10^. To train on on-manifold adversarial examples is generalization and to train on $\epsilon$-away off-manifold adversarial examples is robustness^4^. Following the general properties of information^10^, demonstrates that the results of learning based off informativeness criteria such as entropy and classification margin is better than the results of learning randomly from the train set. Our goal is to estimate the true manifold for every class. We achieve this through maximizing information gain.

Active machine learning facilitates the identifying of the true model through leveraging acquisition function and, in turn, informativeness criteria such as entropy and classification margin. This means that active machine learning is made possible through the quantification of uncertainty. Bayesian inference provides a basis for uncertainty quantification by the means of probability distributions. In discriminative modeling, Prior establishes our prior belief about the plausibility of a function. Posterior belief of parameters would then be given by Bayes’ theorem as

$$\begin{aligned} P(W|D)=\frac{P(D|W)P(W)}{P(D)} \end{aligned}$$

Suppose $\Omega$ is one informativeness critorion, that is, knowing $y=\Omega (x^*)$ improves the accuracy of model to the largest extent compared to all $x \in X$. Then training on $x^*$ achieves the best accuracy. This implies that $x^*$ offers the most amount of information in deciding on model’s decision boundary. Training on adversarial examples selected by such an informativeness criterion is likely to improve adversarial robustness. Selecting low margin instances, that is, instances which share high degrees of fractional dimensionality with more than one class, works to minimize boundary error and in turn, robust error:

$$\begin{aligned} R_{Rob}(f) = R_{Nat}(f)+R_{Bdy}(f) \end{aligned}$$

$$\begin{aligned} Minimize: R_{Bdy}(f) \implies Minimize: R_{Rob}(f) \end{aligned}$$

$$\begin{aligned} Constraint: R_{Nat} \ does\ not\ increase \end{aligned}$$

Above-mentioned minimization objective seeks to estimate unknown parameters pertaining to the model’s concept during training. The effectiveness of the optimization process is impacted by the not deterministic but stochastic nature of the stochastic gradient descent algorithm. The adversarial training procedure can be viewed as a form of active machine learning where the model is able to obtain labels on new points from a heuristic labeler^2^. Low classification margin indicates potential overlap or proximity as veiwed by the model’s concept during training which is likely to maximize information gain of a training method.

## Data Availability

The imlementation of the active machine learning approach to adversarial training is presented at https://github. com/DrMirsadeghi/TheActiveMachineLearningApproachToAdversarialTraining/. The benchmark datasets are publicly available at https://www.cs.toronto.edu/kriz/cifar.html and http://ufldl.stanford. edu/housenumbers/.

## Appendix

### Significance test

In order to test the effect on the active machine learning approach to adversarial training on the accuracy-robustness trade-off of adversarial training against strong PGD and AA adversaries, we formulate a null hypothesis $H_0$ and an alternative hypothesis $H_1$. $H_0$ states the method led to no improvement in the accuracy-robustness trade-off. $H_1$ states that the method yielded improvement in the accuracy-robustness trade-off. We set the significance level $\alpha =0.05$. Hereby, we compute the probability (p-value) that the method yielded improvement as extreme as reported in Table 3 given $H_0$. The p-value is < 0.00001. The result is significant at p < 0.05. Therefore, we are able to favor $H_1$. In conclusion, the active machine learning approach to adversarial training yielded significant improvment to generalization, robustness, and the accuracy-robustness trade-off of adversarial training against strong PGD and AA adversaries.

### Extensive results

**Table 6:** Accuracy (%) and robustness (%) of training methods on CIFAR-10, CIFAR-100, and SVHN. We report the averaged results and confidence intervals of two runs. The performance improvements are reported in bold and italic. Our method confers significant levels of robustness and accuracy upon training methods.

Classifier	Dataset	Method	Accuracy	Robust accuracy		
				FGSM	PGD	AA
ResNet18	CIFAR-10	Standard training	75.40 ± 0.39	3.81 ± 0.19	0.01 ± 0.01	0.00 ± 0.00
		AT	65.95 ± 0.35	39.90 ± 0.29	36.39 ± 0.27	34.51 ± 0.28
		MART	63.68 ± 0.55	42.12 ± 0.40	39.96 ± 0.35	34.55 ± 0.38
		TRADES	65.12 ± 0.78	39.93 ± 0.55	37.86 ± 0.49	33.81 ± 0.49
		Ours (AT)	69.36 ± 0.36 ***(+3.42)***	42.32 ± 0.21 ***(+2.42)***	38.05 ± 0.19 ***(+1.66)***	35.89 ± 0.18 ***(+1.38)***
		Ours (MART)	68.60 ± 0.64 ***(+4.92)***	44.22 ± 0.35 ***(+2.10)***	40.73 ± 0.28 ***(+0.76)***	36.37 ± 0.30 ***(+1.82)***
		Ours (TRADES)	69.66 ± 0.68 ***(+4.54)***	40.75 ± 0.51 ***(+0.82)***	37.97 ± 0.45 ***(+0.11)***	32.88 ± 0.47 **(-0.93)**
	CIFAR-100	Standard training	47.05 ± 0.29	1.19 ± 0.06	0.05 ± 0.01	0.00 ± 0.00
		AT	40.76 ± 0.19	17.49 ± 0.16	15.06 ± 0.15	14.03 ± 0.15
		MART	39.02 ± 0.29	19.93 ± 0.18	18.53 ± 0.17	14.88 ± 0.16
		TRADES	34.10 ± 0.90	18.77 ± 0.51	17.99 ± 0.47	14.35 ± 0.40
		Ours (AT)	41.31 ± 0.27 ***(+0.56)***	17.66 ± 0.18 ***(+0.17)***	14.98 ± 0.20 **(-0.08)**	14.02 ± 0.19 **(-0.02)**
		Ours (MART)	40.78 ± 0.26 ***(+1.76)***	19.36 ± 0.20 **(-0.57)**	17.14 ± 0.22 **(-1.39)**	14.82 ± 0.16 **(-0.07)**
		Ours (TRADES)	36.42 ± 0.73 ***(+2.31)***	18.40 ± 0.37 **(-0.37)**	17.19 ± 0.33 **(-0.80)**	13.01 ± 0.27 **(-1.33)**
	SVHN	Standard training	88.68 ± 0.22	12.91 ± 0.31	2.48 ± 0.13	1.51 ± 0.08
		AT	83.94 ± 0.35	54.96 ± 0.47	45.18 ± 0.35	42.27 ± 0.35
		MART	83.80 ± 0.77	55.41 ± 0.60	48.42 ± 0.51	41.00 ± 0.47
		TRADES	80.23 ± 0.51	53.32 ± 0.50	46.98 ± 0.36	41.51 ± 0.35
		Ours (AT)	86.56 ± 0.27 ***(+2.62)***	59.84 ± 0.55 ***(+4.88)***	48.55 ± 0.35 ***(+3.37)***	45.48 ± 0.32 ***(+3.21)***
		Ours (MART)	86.83 ± 1.01 ***(+3.02)***	60.31 ± 0.78 ***(+4.90)***	51.03 ± 0.59 ***(+2.61)***	43.89 ± 0.52 ***(+2.89)***
		Ours (TRADES)	86.12 ± 0.30 ***(+5.88)***	59.99 ± 0.55 ***(+6.67)***	51.01 ± 0.34 ***(+4.03)***	44.02 ± 0.35 ***(+2.51)***
WRN34	CIFAR-10	Standard training	77.15 ± 0.53	4.35 ± 0.23	0.00 ± 0.00	0.00 ± 0.00
		AT	69.82 ± 0.43	42.37 ± 0.36	38.76 ± 0.32	36.86 ± 0.31
		MART	66.89 ± 0.70	43.96 ± 0.48	41.66 ± 0.43	36.14 ± 0.43
		TRADES	67.78 ± 0.86	41.83 ± 0.65	39.77 ± 0.59	35.59 ± 0.59
		Ours (AT)	73.23 ± 0.42 ***(+3.40)***	45.12 ± 0.29 ***(+2.75)***	40.78 ± 0.25 ***(+2.02)***	38.62 ± 0.25 ***(+1.76)***
		Ours (MART)	72.01 ± 1.22 ***(+5.12)***	47.27 ± 0.73 ***(+3.31)***	43.80 ± 0.62 ***(+2.14)***	38.93 ± 0.60 ***(+2.79)***
		Ours (TRADES)	73.07 ± 0.86 ***(+5.29)***	43.90 ± 0.66 ***(+2.07)***	41.20 ± 0.58 ***(+1.44)***	36.18 ± 0.60 ***(+0.59)***
	CIFAR-100	Standard training	49.92 ± 0.42	1.36 ± 0.08	0.02 ± 0.01	0.00 ± 0.00
		AT	45.40 ± 0.40	20.95 ± 0.26	18.42 ± 0.23	17.11 ± 0.22
		MART	43.90 ± 0.50	23.53 ± 0.30	22.27 ± 0.27	17.85 ± 0.26
		TRADES	37.80 ± 1.12	21.10 ± 0.62	20.28 ± 0.57	16.36 ± 0.49
		Ours (AT)	47.28 ± 0.33 ***(+1.88)***	21.88 ± 0.24 ***(+0.93)***	18.83 ± 0.24 ***(+0.41)***	17.40 ± 0.23 ***(+0.29)***
		Ours (MART)	47.85 ± 0.36 ***(+3.95)***	24.08 ± 0.24 ***(+0.55)***	21.78 ± 0.23 **(-0.49)**	18.61 ± 0.22 ***(+0.76)***
		Ours (TRADES)	41.72 ± 0.91 ***(+3.92)***	21.58 ± 0.49 ***(+0.48)***	20.29 ± 0.44 ***(+0.01)***	15.67 ± 0.38 **(-0.68)**
	SVHN	Standard training	89.92 ± 0.33	14.45 ± 0.36	2.70 ± 0.14	1.70 ± 0.10
		AT	85.90 ± 0.33	59.64 ± 0.60	47.77 ± 0.43	44.30 ± 0.41
		MART	86.80 ± 0.34	63.40 ± 0.64	52.55 ± 0.43	43.99 ± 0.39
		TRADES	76.42 ± 1.40	59.36 ± 1.55	48.43 ± 0.88	41.08 ± 0.69
		Ours (AT)	87.94 ± 0.26 ***(+2.04)***	63.99 ± 0.73 ***(+4.35)***	50.76 ± 0.45 ***(+2.99)***	46.58 ± 0.44 ***(+2.28)***
		Ours (MART)	89.88 ± 0.29 ***(+3.08)***	68.03 ± 0.78 ***(+4.63)***	55.75 ± 0.47 ***(+3.20)***	46.58 ± 0.47 ***(+2.58)***
		Ours (TRADES)	85.04 ± 1.22 ***(+8.62)***	60.59 ± 1.10 ***(+1.23)***	52.41 ± 0.81 ***(+3.98)***	45.07 ± 0.72 ***(+3.98)***

**Table 7:** F1-robust (%) and computation time (hours) of training methods on CIFAR-10, CIFAR-100, and SVHN. F1-robust is the harmonic mean between robustness and natural accuracy. The performance improvements are reported in bold and italic. The additional computation time is reported in bold. Our method consistently improves F1-robust of adversarial training methods. This finding is significant in addressing the accuracy-robustness trade-off with adversarial training.

Classifier	Dataset	Method	F1-robust			Compute
			FGSM	PGD	AA	
ResNet18	CIFAR-10	Standard training	5.91 ± 0.28	0.01 ± 0.01	0.00 ± 0.00	13.93 ± 0.12
		AT	45.87 ± 0.32	42.70 ± 0.31	40.95 ± 0.31	18.42 ± 0.17
		MART	47.22 ± 0.46	45.35 ± 0.42	40.45 ± 0.45	18.96 ± 0.10
		TRADES	45.73 ± 0.78	39.93 ± 0.55	37.86 ± 0.49	33.81 ± 0.49
		Ours (AT)	48.94 ± 0.25 ***(+3.07)***	45.11 ± 0.22 ***(+2.41)***	43.11 ± 0.21 ***(+2.16)***	18.94 ± 0.13 ***(+0.53)***
		Ours (MART)	50.39 ± 0.45 ***(+3.17)***	47.35 ± 0.37 ***(+2.00)***	43.39 ± 0.39 ***(+2.94)***	19.12 ± 0.02 ***(+0.17)***
		Ours (TRADES)	47.67 ± 0.62 ***(+1.94)***	45.16 ± 0.57 ***(+1.29)***	40.32 ± 0.59 ***(+0.19)***	18.91 ± 0.02 **(-14.9)**
	CIFAR-100	Standard training	1.54 ± 0.08	0.06 ± 0.02	0.00 ± 0.00	0.52 ± 0.00
		AT	19.79 ± 0.18	17.28 ± 0.16	16.19 ± 0.16	10.15 ± 0.02
		MART	22.04 ± 0.20	20.63 ± 0.19	16.93 ± 0.18	10.41 ± 0.17
		TRADES	20.39 ± 0.90	18.77 ± 0.51	17.99 ± 0.47	14.35 ± 0.40
		Ours (AT)	20.03 ± 0.21 ***(+0.24)***	17.24 ± 0.22 **(-0.04)**	16.23 ± 0.22 ***(+0.04)***	11.85 ± 0.08 ***(+1.69)***
		Ours (MART)	21.68 ± 0.22 **(-0.36)**	19.44 ± 0.24 **(-1.19)**	17.03 ± 0.19 ***(+0.10)***	11.84 ± 0.32 ***(+1.43)***
		Ours (TRADES)	20.26 ± 0.43 **(-0.13)**	19.05 ± 0.38 **(-0.58)**	14.78 ± 0.32 **(-1.21)**	10.52 ± 0.15 ***(+3.00)***
	SVHN	Standard training	20.75 ± 0.47	4.35 ± 0.22	2.67 ± 0.14	16.06 ± 0.68
		AT	64.24 ± 0.47	56.03 ± 0.39	53.38 ± 0.38	19.79 ± 0.63
		MART	64.61 ± 0.70	58.88 ± 0.62	52.20 ± 0.60	17.42 ± 4.47
		TRADES	61.6 ± 0.51	53.32 ± 0.50	46.98 ± 0.36	41.51 ± 0.35
		Ours (AT)	69.01 ± 0.51 ***(+4.77)***	59.93 ± 0.37 ***(+3.89)***	57.22 ± 0.35 ***(+3.84)***	20.07 ± 0.07 ***(+0.28)***
		Ours (MART)	69.58 ± 0.87 ***(+4.97)***	62.23 ± 0.74 ***(+3.35)***	55.98 ± 0.68 ***(+3.77)***	20.07 ± 0.15 ***(+2.65)***
		Ours (TRADES)	68.96 ± 0.50 ***(+7.36)***	61.87 ± 0.36 ***(+5.53)***	55.73 ± 0.37 ***(+4.25)***	19.82 ± 0.11 **(-21.69)**
WRN34	CIFAR-10	Standard training	6.81 ± 0.34	0.00 ± 0.00	0.00 ± 0.00	3.27 ± 0.02
		AT	49.12 ± 0.41	45.88 ± 0.38	44.14 ± 0.37	85.84 ± 0.91
		MART	49.68 ± 0.57	47.71 ± 0.52	42.74 ± 0.53	75.91 ± 18.36
		TRADES	48.11 ± 0.86	41.83 ± 0.65	39.77 ± 0.59	35.59 ± 0.59
		Ours (AT)	52.51 ± 0.35 ***(+3.39)***	48.67 ± 0.31 ***(+2.80)***	46.71 ± 0.30 ***(+2.57)***	75.60 ± 1.41 **(-10.24)**
		Ours (MART)	54.07 ± 0.91 ***(+4.38)***	51.13 ± 0.81 ***(+3.42)***	46.79 ± 0.80 ***(+4.05)***	75.72 ± 1.37 **(-0.19)**
		Ours (TRADES)	51.44 ± 0.79 ***(+3.33)***	49.06 ± 0.73 ***(+2.76)***	44.41 ± 0.75 ***(+1.94)***	74.72 ± 1.35 ***(+39.13)***
	CIFAR-100	Standard training	20.75 ± 0.47	4.35 ± 0.22	2.67 ± 0.14	16.06 ± 0.68
		AT	64.24 ± 0.47	56.03 ± 0.39	53.38 ± 0.38	19.79 ± 0.63
		MART	64.61 ± 0.70	58.88 ± 0.62	52.20 ± 0.60	17.42 ± 4.47
		TRADES	61.6 ± 0.51	53.32 ± 0.50	46.98 ± 0.36	41.51 ± 0.35
		Ours (AT)	69.01 ± 0.51 ***(+4.77)***	59.93 ± 0.37 ***(+3.89)***	57.22 ± 0.35 ***(+3.84)***	20.07 ± 0.07 ***(+0.28)***
		Ours (MART)	69.58 ± 0.87 ***(+4.97)***	62.23 ± 0.74 ***(+3.35)***	55.98 ± 0.68 ***(+3.77)***	20.07 ± 0.15 ***(+2.65)***
		Ours (TRADES)	68.96 ± 0.50 ***(+7.36)***	61.87 ± 0.36 ***(+5.53)***	55.73 ± 0.37 ***(+4.25)***	19.82 ± 0.11 **(-21.69)**
	SVHN	Standard training	23.16 ± 0.54	4.75 ± 0.24	3.04 ± 0.18	23.40 ± 0.55
		AT	68.60 ± 0.57	58.99 ± 0.46	55.91 ± 0.45	79.15 ± 6.47
		MART	71.74 ± 0.58	63.37 ± 0.45	55.95 ± 0.44	83.54 ± 1.23
		TRADES	64.78 ± 1.40	59.36 ± 1.55	48.43 ± 0.88	41.08 ± 0.69
		Ours (AT)	72.60 ± 0.60 ***(+4.00)***	62.30 ± 0.44 ***(+3.31)***	58.69 ± 0.44 ***(+2.77)***	86.48 ± 3.58 ***(+7.33)***
		Ours (MART)	76.27 ± 0.64 ***(+4.53)***	67.17 ± 0.48 ***(+3.80)***	59.41 ± 0.50 ***(+3.45)***	88.96 ± 1.96 ***(+5.42)***
		Ours (TRADES)	69.05 ± 1.18 ***(+4.27)***	62.76 ± 0.99 ***(+6.29)***	56.45 ± 0.92 ***(+6.37)***	86.56 ± 0.25 ***(+45.48)***

**Table 8:** We provide best checkpoints to address the concern of robust overfitting.

Classifier	Dataset	Method	FGSM		PGD		AA	
			Best	Last	Best	Last	Best	Last
ResNet18	CIFAR-10	Standard training	7.81	5.08	0.29	0.00	0.00	0.00
		AT	43.95	43.16	40.62	38.67	38.96	36.91
		MART	47.36	43.65	45.51	41.21	39.94	36.82
		TRADES	46.00	42.87	43.36	40.33	39.55	37.50
		Ours (AT)	45.70 ***(+1.76)***	41.60	41.02 ***(+0.39)***	37.60	38.87 **(-0.10)**	35.35
		Ours (MART)	47.95 ***(+0.59)***	46.39	44.82 **(-0.68)**	41.60	40.14 ***(+0.20)***	37.79
		Ours (TRADES)	46.48 ***(+0.49)***	44.14	42.77 **(-0.59)**	40.72	37.79 **(-1.76)**	35.74
	CIFAR-100	Standard training	2.54	1.86	0.68	0.20	0.00	0.00
		AT	20.80	17.68	18.36	14.94	17.19	13.87
		MART	23.05	19.43	21.48	18.36	17.38	15.82
		TRADES	22.75	22.75	21.97	21.97	17.48	16.41
		Ours (AT)	21.00 ***(+0.20)***	16.31	18.46 ***(+0.10)***	13.87	17.38 ***(+0.20)***	12.50
		Ours (MART)	22.56 **(-0.49)**	17.38	20.61 **(-0.88)**	15.33	17.19 **(-0.20)**	13.18
		Ours (TRADES)	21.97 **(-0.78)**	18.95	20.41 **(-1.56)**	17.38	15.92 **(-1.56)**	13.38
	SVHN	Standard training	19.63	15.14	5.86	4.00	3.71	2.93
		AT	62.21	56.84	49.61	46.97	46.29	44.04
		MART	61.33	55.76	53.61	47.27	46.09	40.92
		TRADES	60.25	54.88	52.34	47.85	45.61	42.38
		Ours (AT)	66.99 ***(+4.79)***	64.16	53.61 ***(+4.00)***	51.76	49.41 ***(+3.12)***	47.46
		Ours (MART)	67.48 ***(+6.15)***	66.80	56.74 ***(+3.12)***	54.49	49.12 ***(+3.03)***	46.78
		Ours (TRADES)	71.48 ***(+11.23)***	71.48	55.96 ***(+3.61)***	53.71	49.02 ***(+3.42)***	46.58
WRN34	CIFAR-10	Standard training	9.86	6.74	0.10	0.00	0.00	0.00
		AT	46.58	43.85	43.46	41.02	41.31	38.38
		MART	49.51	46.88	47.75	42.77	42.19	37.60
		TRADES	47.75	47.07	44.73	43.85	40.62	39.94
		Ours (AT)	48.83 ***(+2.25)***	45.90	44.34 ***(+0.88)***	41.60	42.19 ***(+0.88)***	38.67
		Ours (MART)	52.64 ***(+3.12)***	49.32	48.73 ***(+0.98)***	44.04	44.04 ***(+1.86)***	39.84
		Ours (TRADES)	49.41 ***(+1.66)***	47.36	47.17 ***(+2.44)***	44.14	42.09 ***(+1.46)***	39.26
	CIFAR-100	Standard training	2.73	1.37	0.39	0.10	0.10	0.10
		AT	24.90	23.63	21.58	20.21	19.92	18.85
		MART	27.34	25.39	25.68	24.12	21.19	18.85
		TRADES	26.46	25.20	25.29	24.80	20.61	20.21
		Ours (AT)	26.17 ***(+1.27)***	20.90	23.05 ***(+1.46)***	17.38	21.48 ***(+1.56)***	15.92
		Ours (MART)	28.12 ***(+0.78)***	22.07	25.49 **(-0.20)**	18.75	21.97 ***(+0.78)***	16.70
		Ours (TRADES)	25.39 **(-1.07)**	23.34	23.93 **(-1.37)**	21.88	19.53 **(-1.07)**	17.29
	SVHN	Standard training	21.78	12.79	5.66	1.37	4.10	0.68
		AT	70.41	63.67	53.71	51.46	48.83	48.14
		MART	75.68	64.75	59.47	52.93	50.39	45.12
		TRADES	78.61	58.89	57.42	51.37	48.83	45.31
		Ours (AT)	83.40 ***(+12.99)***	69.63	68.07 ***(+14.36)***	49.22	52.15 ***(+3.32)***	45.51
		Ours (MART)	83.30 ***(+7.62)***	83.30	65.82 ***(+6.35)***	60.06	51.86 ***(+1.46)***	46.78
		Ours (TRADES)	79.10 ***(+0.49)***	59.38	60.84 ***(+3.42)***	53.03	50.39 ***(+1.56)***	46.88

**Table 9:** Accuracy (%) and robustness (%) of training methods on Tiny-Imagenet-200 dataset. The performance improvements are reported in bold and italic. Our method confers significant levels of robustness and accuracy upon training methods.

Classifier	Method	Accuracy	Robust accuracy		
			FGSM	PGD	AA
ResNet18	Standard training	28.85 ± 0.31	0.18 ± 0.02	0.00 ± 0.00	0.00 ± 0.00
	AT	18.20 ± 0.17	5.75 ± 0.09	4.83 ± 0.08	4.03 ± 0.08
	MART	17.28 ± 0.21	7.79 ± 0.11	7.35 ± 0.11	4.40 ± 0.08
	TRADES	21.46 ± 0.20	6.11 ± 0.10	5.22 ± 0.09	4.12 ± 0.08
	Ours (AT)	22.00 ± 0.20 ***(+3.79)***	8.51 ± 0.12 ***(+2.76)***	7.09 ± 0.10 ***(+2.25)***	6.33 ± 0.09 ***(+2.30)***
	Ours (MART)	21.58 ± 0.22 ***(+4.30)***	9.72 ± 0.13 ***(+1.93)***	9.06 ± 0.12 ***(+1.71)***	5.68 ± 0.10 ***(+1.29)***
	Ours (TRADES)	26.60 ± 0.20 ***(+5.14)***	7.07 ± 0.11 ***(+0.96)***	5.90 ± 0.10 ***(+0.68)***	4.53 ± 0.09 ***(+0.41)***
WRN34	Standard training	28.81 ± 0.39	0.29 ± 0.02	0.00 ± 0.00	0.00 ± 0.00
	AT	18.42 ± 0.20	6.07 ± 0.10	5.15 ± 0.09	4.35 ± 0.08
	MART	18.01 ± 0.21	8.39 ± 0.14	8.00 ± 0.13	4.86 ± 0.10
	TRADES	22.10 ± 0.20	6.65 ± 0.11	5.68 ± 0.09	4.53 ± 0.08
	Ours (AT)	22.23 ± 0.23 ***(+3.81)***	8.83 ± 0.14 ***(+2.76)***	7.40 ± 0.11 ***(+2.25)***	6.63 ± 0.11 ***(+2.28)***
	Ours (MART)	23.03 ± 0.22 ***(+5.02)***	10.42 ± 0.13 ***(+2.03)***	9.83 ± 0.12 ***(+1.82)***	6.34 ± 0.11 ***(+1.49)***
	Ours (TRADES)	27.78 ± 0.21 ***(+5.68)***	8.17 ± 0.14 ***(+1.51)***	6.70 ± 0.12 ***(+1.02)***	5.28 ± 0.11 ***(+0.75)***

**Table 10:** F1-robust (%) and computation time (hours) of training methods on Tiny-Imagenet-200. F1-robust is the harmonic mean between robustness and natural accuracy. The performance improvements are reported in bold and italic. The additional computation time is reported in bold. Our method consistently improves F1-robust of adversarial training methods. This finding is significant in addressing the accuracy-robustness trade-off with adversarial training.

Classifier	Method	F1-robust			Compute
		FGSM	PGD	AA	
ResNet18	Standard training	0.21 ± 0.02	0.00 ± 0.00	0.00 ± 0.00	2.20 ± 0.05
	AT	6.13 ± 0.10	5.17 ± 0.09	4.34 ± 0.08	22.97 ± 3.59
	MART	8.18 ± 0.12	7.74 ± 0.12	4.70 ± 0.09	24.39 ± 0.06
	TRADES	6.63 ± 0.20	6.11 ± 0.10	5.22 ± 0.09	4.12 ± 0.08
	Ours (AT)	9.12 ± 0.13 ***(+2.99)***	7.67 ± 0.11 ***(+2.50)***	6.87 ± 0.10 ***(+2.53)***	22.38 ± 0.08 ***(+-0.59)***
	Ours (MART)	10.33 ± 0.14 ***(+2.15)***	9.66 ± 0.13 ***(+1.92)***	6.18 ± 0.10 ***(+1.47)***	25.10 ± 3.10 ***(+0.72)***
	Ours (TRADES)	7.84 ± 0.12 ***(+1.21)***	6.57 ± 0.11 ***(+0.89)***	5.10 ± 0.10 ***(+0.59)***	26.76 ± 0.23 ***(+3.39)***
WRN34	Standard training	0.34 ± 0.03	0.01 ± 0.00	0.00 ± 0.00	11.16 ± 0.30
	AT	6.46 ± 0.11	5.51 ± 0.10	4.68 ± 0.09	90.78 ± 0.29
	MART	8.82 ± 0.14	8.43 ± 0.14	5.20 ± 0.11	107.53 ± 2.22
	TRADES	7.21 ± 0.20	6.65 ± 0.11	5.68 ± 0.09	4.53 ± 0.08
	Ours (AT)	9.46 ± 0.15 ***(+3.00)***	7.99 ± 0.13 ***(+2.47)***	7.19 ± 0.12 ***(+2.51)***	103.09 ± 4.35 ***(+12.31)***
	Ours (MART)	11.12 ± 0.14 ***(+2.31)***	10.50 ± 0.13 ***(+2.06)***	6.92 ± 0.12 ***(+1.72)***	114.94 ± 1.28 ***(+7.42)***
	Ours (TRADES)	9.05 ± 0.15 ***(+1.84)***	7.50 ± 0.13 ***(+1.32)***	5.95 ± 0.12 ***(+0.98)***	158.73 ± 46.67 ***(+52.35)***

**Table 11:** Tiny-Imagenet-200 dataset: We provide best checkpoints to address the concern of robust overfitting.

Classifier	Method	FGSM		PGD		AA	
		Best	Last	Best	Last	Best	Last
ResNet18	Standard training	0.68	0.20	0.10	0.00	0.00	0.00
	AT	7.62	6.15	6.64	5.37	5.66	4.49
	MART	9.96	8.20	9.38	7.91	6.05	4.98
	TRADES	8.01	7.03	6.84	5.96	5.47	4.79
	Ours (AT)	10.55 ***(+2.93)***	8.30	8.69 ***(+2.05)***	7.13	7.81 ***(+2.15)***	6.54
	Ours (MART)	12.89 ***(+2.93)***	11.23	11.91 ***(+2.54)***	9.18	7.13 ***(+1.07)***	5.86
	Ours (TRADES)	9.08 ***(+1.07)***	8.59	7.52 ***(+0.68)***	7.23	6.05 ***(+0.59)***	5.86
WRN34	Standard training	0.78	0.20	0.10	0.00	0.00	0.00
	AT	8.30	7.03	7.32	6.05	6.15	4.98
	MART	10.45	10.06	9.86	9.77	6.25	5.96
	TRADES	8.89	7.23	7.62	5.86	5.76	4.59
	Ours (AT)	10.55 ***(+2.25)***	10.45	8.98 ***(+1.66)***	8.69	8.20 ***(+2.05)***	7.71
	Ours (MART)	12.50 ***(+2.05)***	10.94	11.52 ***(+1.66)***	10.64	7.81 ***(+1.56)***	6.74
	Ours (TRADES)	9.57 ***(+0.68)***	9.18	8.40 ***(+0.78)***	7.52	6.64 ***(+0.88)***	5.86

## Declarations

### Extensibility

We implement the active machine learning approach to adversarial training with multiple adversarial training methods such as Standard Adversarial Training (AT), Misclassification Aware adveRsarial Training (MART), and Theoretically Principled Trade-off between Accuracy and Robustness (TRADES). That said, the method is highly modular and integratable into other adversarial training methods. This design makes the method straightforward to extend to new settings where different operations may be desired.
